# Exploring the Environmental Exposure to Methoxychlor, α-HCH and Endosulfan–sulfate Residues in Lake Naivasha (Kenya) Using a Multimedia Fate Modeling Approach

**DOI:** 10.3390/ijerph17082727

**Published:** 2020-04-15

**Authors:** Yasser Abbasi, Chris M. Mannaerts

**Affiliations:** Department of Water Resources, Faculty of Geo-Information Science and Earth Observation, University of Twente, Hengelosestraat 99, 7514 AE Enschede, The Netherlands; c.m.m.mannaerts@utwente.nl

**Keywords:** organochlorine pesticides, fate modeling, QWASI model, multimedia, passive sampling

## Abstract

Distribution of pesticide residues in the environment and their transport to surface water bodies is one of the most important environmental challenges. Fate of pesticides in the complex environments, especially in aquatic phases such as lakes and rivers, is governed by the main properties of the contaminants and the environmental properties. In this study, a multimedia mass modeling approach using the Quantitative Water Air Sediment Interaction (QWASI) model was applied to explore the fate of organochlorine pesticide residues of methoxychlor, α-HCH and endosulfan–sulfate in the lake Naivasha (Kenya). The required physicochemical data of the pesticides such as molar mass, vapor pressure, air–water partitioning coefficient (K_AW_), solubility, and the Henry’s law constant were provided as the inputs of the model. The environment data also were collected using field measurements and taken from the literature. The sensitivity analysis of the model was applied using One At a Time (OAT) approach and calibrated using measured pesticide residues by passive sampling method. Finally, the calibrated model was used to estimate the fate and distribution of the pesticide residues in different media of the lake. The result of sensitivity analysis showed that the five most sensitive parameters were K_OC_, logKow, half-life of the pollutants in water, half-life of the pollutants in sediment, and K_AW_. The variations of outputs for the three studied pesticide residues against inputs were noticeably different. For example, the range of changes in the concentration of α-HCH residue was between 96% to 102%, while for methoxychlor and endosulfan-sulfate it was between 65% to 125%. The results of calibration demonstrated that the model was calibrated reasonably with the R^2^ of 0.65 and RMSE of 16.4. It was found that methoxychlor had a mass fraction of almost 70% in water column and almost 30% of mass fraction in the sediment. In contrast, endosulfan–sulfate had highest most fraction in the water column (>99%) and just a negligible percentage in the sediment compartment. α-HCH also had the same situation like endosulfan–sulfate (e.g., 99% and 1% in water and sediment, respectively). Finally, it was concluded that the application of QWASI in combination with passive sampling technique allowed an insight to the fate process of the studied OCPs and helped actual concentration predictions. Therefore, the results of this study can also be used to perform risk assessment and investigate the environmental exposure of pesticide residues.

## 1. Introduction

The contamination of water bodies by pesticide residues that can originate from agricultural application is considered as one of the most important environmental issues [1,2]. The residues of the applied pesticides can be transported using surface runoff and pollute the water resources. This issue could be influenced by several factors such as physicochemical properties of the pesticides, topography, soil properties and weather conditions [3]. Among different environmental compartments, surface water resources are considered as important ecosystems that offer a useful environmental service for humans and nature. However, it is a fact that these sources are very sensitive as the human activities cause pollution by chemical emissions [4,5,6].

When the chemicals are emitted into the environment, their fate and distribution in the multimedia environments (e.g., aquatic phases such as lakes) is governed by the main properties of the contaminants such as basic features (e.g., melting point, vapor pressure, partitioning coefficients between soil, sediment and water) [7]. The natural processes can also change the interaction of the chemicals among environmental compartments and cause complexity in predicting their pathways by which the chemicals could enter. Consequently, it is more difficult to study the environmental exposure to the pollutants, which depends on their fate in the environment [8]. The pollutant properties that control the tendency of the pollutants to be transported among different phases are important in the assessment of the pollutants behavior in the environment. This information is the initial input for the models to describe the fate and variations of the chemicals. 

By applying the mathematical equations, it is possible to explain the partitioning, variations of the chemicals and their movements. For this aim, the chemical properties of the pollutants’ variations such as their movements to different media and tendency of partitioning, the mechanisms of chemical loss, the exchange with other media, and their persistency have to be considered. The importance of any one of these parameters and their influence on the chemical’s fate can also be evaluated using the sensitivity analysis [9]. There is a series of models with different levels of complexity that are categorized as Level I, II, and III, which help to fully understand how the properties of the pollutants as well as the environmental features can affect the fate and conveyance of the chemicals. The main features of these models have been described by Mackay [10]. Level I explains the equilibrium dispersion of a specific amount of the pollutant among various environmental phases. Level II model applies the effect of half-life of the pollutant in different environmental compartments. The Level III model, which is the most complex level, consists of all the procedures that affect the transport and fate of the pollutants in an actual environment. In summary, these fugacity models are easy to understand and apply for the assessment of the long-term variations of chemicals [11].

With regard to the difficulty of studying the chemical variations in the environment (e.g., air, water, soil, sediment and biota), using multimedia models is necessary to estimate the fate and transfer of the pesticides [12]. The chemicals that are nonreactive and persistent against degradation can remain in the environment for a long time and make drastic disasters by entering and accumulating in the environment. For such pollutants, it is proposed to use models with a multimedia mass balance approach [11]. The fugacity models [10] that are used for this aim can help by simplifying the calculations. Among different multimedia models, the fugacity model by Mackay [13] has been used successfully in many case studies to evaluate the fate of chemicals [12,14,15]. Many of the studies quantify the fate and exchange of the chemicals in the environment by considering the steady or unsteady conditions [16,17,18]. The Quantitative Water Air Sediment Interaction (QWASI) fugacity model [19] is one of the models that has been used to explore the chemical variations (e.g., pesticides and heavy metals) by many researchers [12]. 

The QWASI model was established based on the fugacity perception that has been widely and freely available for the fate modeling of chemicals in the environment [20]. The QWASI model assumes a well-mixed aquatic environment as well as mass balance procedure. In this model, the mass balance equations have been applied to establish a steady state condition for both sediment and aquatic media. Moreover, this concept is applied for chemicals’ contents using the fugacity model. It is notable that the model can also be modified to one and two order differential equations for the dynamic situations, and then be calculated numerically or analytically. The models are easily understood and interpreted because the procedures are presented using the fugacity concept that allows interpretation of the pollutant’s diffusion, reaction and advection conveyance [20]. 

In this study, the multimedia mass modeling approach was applied to explore the fate of organochlorine pesticides residues in the lake Naivasha (Kenya). Naivasha lake is one of the most important fresh water resources in the region. It provides irrigation water for most of the riparian farms in the area as well as the fresh water for domestic drinking and supports a variety of wildlife around the lake wetland ecosystem [21]. However, the previous studies showed the residues of some organochlorine pesticides (OCPs) in the water resources of Naivasha [21,22]. Based on the Secretariat of the Stockholm Convention on Persistent Organic Pollutants, these pesticides are categorized as the persistent chemicals [23] that have residues that can remain in the environment for a long time. In addition to the studies mentioned earlier, several recent researches have applied QWASI or other kinds of modelling approaches [24,25,26,27,28,29]. In most of these studies, fugacity models have been applied to explore the fate of chemical residues in different media. In this study, also the multimedia modeling approach in combination with measured low concentrations of pesticide residues using the passive sampling method combined with laboratory gas chromatography of the lake Naivasha water was applied, which is the novelty of this study, with respect to other studies for predicting the distribution and fate of pesticide residues. Finally, due to the high concentration of Lindane residue (e.g., α-HCH), endosulfan–sulfate and methoxychlor [22], it was decided to explore the fate of these residues in the aquatic environment of Naivasha.

## 2. Materials and Methods

### 2.1. Data Acquisition 

**Figure 1:**
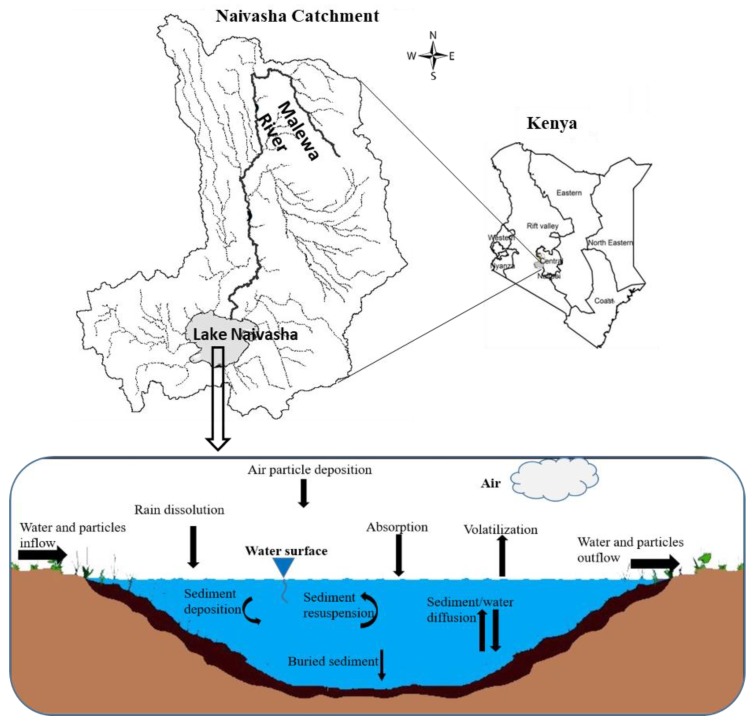
Schematic profile of the lake Naivasha and representation of the QWASI model (reproduced partly from Whelan (2013) [28]) for different environmental compartments.

In this study, the fugacity multimedia model of QWASI [19] was used to evaluate the environmental exposure to α-HCH, endosulfan–sulfate and methoxychlor pesticide residues. This model is a steady state non-equilibrium (Level III) multi-media fate and transport model that has been designed specifically to represent processes operating in lakes [30]. Chemical behavior is represented in the model using fugacity concepts [10]. The processes considered by the model and the studied area are depicted schematically in Figure 1.

The model needs the physicochemical data of the pollutants as well as the environmental properties data as inputs information to calculate the level of pollution in every environmental compartment. The physicochemical properties of α-HCH, methoxychlor and endosulfan-sulfate are presented in Table 1 and Table 2. After providing information of molar mass, vapor pressure, solubility of the chemicals, and the temperature, the model is able to calculate the Henry’s law constant as well as the air–water partitioning coefficient (K_AW_). Based on different studies [20,31], the measured *K_AW_* amounts that are reported in the literatures are significantly different than each other among which the analytical technique established by Xu and Kropscott [31] is the most suitable method [20]. The Henry’s law constant and the air–water partitioning coefficient (K_AW_) were calculated as follows:

H= P/S × M(1)

where H is Henry’s law constant, P is vapor pressure (Pa), S is the chemical solubility in water (mg/L), and M is the molar mass (g/mol). Consequently, the K_AW_ coefficient was calculated by considering the Henry’s constant and the temperature as follows:

K_AW_ = H/RT (2)

where H is Henry’s law constant, R is the gas constant (8.314 J K^−1^ mol^−1^) and T is the temperature at the Kelvin scale (K). Mackay et al. [20] also used the next equation to calculate K_AW_ at a specific temperature:

(3) $$K_{AW}\left(T\right)=K_{AW}\left(T_{r}\right).exp\left(\frac{\Delta U_{AW}}{R}\left(\frac{1}{T_{r}}-\frac{1}{T}\right)\right)$$

with Δ*U*_AW_ = 92.7 kJ/mol. 

The next coefficient that is used by the model is the organic carbon–water partition coefficient (K_OC_). Karickhoff [32] found that this factor could almost totally govern the sorption capability of sediment. Consequently, the partitioning tendency between water and sediment, which is represented as K_OC_ coefficient, can be connected to the octanol–water partitioning coefficient (K_OW_). He developed the relationship between these two coefficients using experiments with different soil organic matter percentage and pollutants that had different K_OW_ [33]. Then the K_OC_ factor is calculated from K_OW_ as follows:

K_OC_ = 0.41K_ow_ (4)

Additionally, the model takes the half-lives of chemicals into account both in the aquatic and sediment environments to evaluate their fate. The half- life of every individual chemical depends on both the physicochemical properties of the pollutant and the environmental properties. The model estimates the reaction half-life at different temperatures using the activation energy (J/mol) in a desired temperature (°C) as this:

(5) $$\tau_{T}=\tau_{298.15}exp\left[\frac{E_{A}}{R}\;\left(\frac{1}{T}-\frac{1}{298.15}\right)\right]$$

where E_A_ is the activation energy (J/mol), T is the temperature (converted from °C to K), and R is gas constant (8.314 J K^−1^ mol^−1^). 

In addition to the chemicals data, the QWASI model needs the environment data to predict the fate of pollutants. The details of the environmental input data (Table 2) are explained in the following section. 

### 2.2. Environment Characteristics 

Lake Naivasha is one of the fresh water resources in the Eastern Rift valley of Kenya with a latitude of 00°46’ to 00°52’ and longitude 36°15’ to 36°25’ in zone 37S UTM (Figure 1). The most important streams in the Lake Naivasha basin are Malewa, Gilgil and Karati rivers. The main inflow to the lake is Malewa river, which provides up to 80% of the total inflow into the Lake [34], but there is not any identifiable outlet from the lake. Another input to the lake comes from the rainfall, which occurs from March to June as the long wet season and from October to early December as the short wet season. The dry months also start from December to February and from July to September, but these durations may change over the years. Agriculture is one of the major parts of the basin that can cause a high potential of pollution in the water resources of the region by using agrochemicals.

The main environmental parameters of the lake Naivasha as inputs of QWASI model are presented in Table 2. Some of these parameters such as the lake dimensions, inflow, suspended sediments, and organic carbon content were measured in the field for this study, but there are several studies also that have already reported environmental parameters of the lake Naivasha [35,36,37]. The volume of the lake was determined using remote sensing data for the lake surface area, bathymetric data and geographic information system (e.g., GIS tools). It is obvious that the depth of the lake changes during the dry and wet seasons. However, in this study, we used the average depth of the lake during the sampling in June–July 2016.

The thickness of the active sediment layer is also a required parameter as an environmental input for the model. This parameter can be changed both temporally and spatially and is difficult to directly measure. Therefore, the initial amount of this input data was selected as the default value in the model and finally was fitted in the calibration process. The concentration of the solids was measured during the field data collection using a 0.45-µm filter paper. For the remaining parameters such as aerosol deposition, volatilization, sedimentation and sediment resuspension, the initial amounts in the model were used, then they were modified during the calibration process.

### 2.3. Pesticides Properties 

In this study, the fate of three kinds of pesticides residues was explored. Lindane is an insecticide for protecting fruits, vegetables and animals against the insects. The isomers of Lindane (e.g., α-HCH, β-HCH, γ-HCH, δ-HCH) can remain in the environment for a long time and are considered as persistent organic pollutants (POPs) by Stockholm Convention on Persistent Organic Pollutants [38]. The environmental properties and interchanges between this pesticide and the environment can influence its fate. The environmental fate of α-HCH is governed by environmental effects and its interactions with the environment as well as its inherent properties (e.g., physical and chemical) [39]. Based on environmental conditions, methoxychlor also can have a half-life of less than 5 h to several months [40]. For instance, the sediment has a half-life of 28 days and more than 100 days in anaerobic and aerobic conditions, respectively [40]. These properties allow the methoxychlor to remain in the environment for a long time and enter the water bodies via different ways such as wind and runoff. Endosulfan–sulfate also, as another POP, has high acute toxicity and can remain in the environment [41]. However, the fate of endosulfan–sulfate also, like other chemicals, is up to the environmental conditions. When it is emitted to the water resources, its residues can be adsorbed by the suspended particles while in the soil its fate is governed by the K_OC_ value, which has a slow movement [41]. 

In this study, the physicochemical properties of the mentioned pesticides were taken in to account for modeling their fate. It is noticeable that as these parameters are variable and direct measuring in the field is almost impossible, the initial values in the literatures were used, and then based on the measured concentrations of the pesticide residues were revised. Moreover, the concentration of the pesticide residues in the lake and the rivers that inflow to the lake also was measured using the passive sampling method followed by laboratory gas chromatography (GC-ECD and GC-MSMS) [22]. In addition to the concentrations in water, some reference samplers were used to measure the pesticide’s concentration in the air that showed the pesticide’s content in the air was below detection limit. Therefore, in this study, we assumed that the concentrations of the pesticides in the air were zero as the model input. 

### 2.4. Sensitivity Analysis and Calibration 

Usually the input data for the modeling process are subjected to uncertainties. Therefore, the effect of the uncertainty of the data was explored using sensitivity analysis [42] for which most influencing parameters were selected carefully. For this aim, the sensitivity analysis using one at a time (OAT) approach was conducted for evaluating the chemical and environmental parameters [30,43]. In this method, the values of desired parameters were changed gradually and their effect on the results was explored. The magnitudes of the yield parameters as Y and the input parameters as X were considered and resulted as the S-matrix below [20]:

(6) $$S=\frac{\delta \ln\left(y\right)}{\delta \ln\left(x\right)}=\frac{\left(\frac{dy}{y}\right)}{\left(\frac{dx}{x}\right)}\sim \frac{\left(\frac{\Delta y}{y}\right)}{\left(\frac{\Delta x}{x}\right)}$$

Where ∆x/x is the partial changes in the input data, and ∆y/y represents the fractional change in the result. It is noticeable that the results can be positive or negative that explain if changing the inputs can increase or decrease the outputs. 

Moreover, as the initial values of the input data are mostly different from the actual values to match the model with the measured data, the results can contain an error that requires a suitable calibration to decrease the uncertainty of the outputs. Therefore, the model was calibrated based on the most sensitive parameters. The values of the parameters were adjusted until the best fit between measured and simulated results was observed (Table 1 and Table 2). The average of the calibrated model’s estimates was compared to the measured concentrations graphically. In addition to this, the results of the calibrations were evaluated using the objective functions R-squared (R^2^) and root mean square error (RMSE). These two functions show how well the model is calibrated as the more R^2^ is close to 1 and RMSE is smaller, the better the model has been calibrated.

## 3. Results and Discussion

**Figure 2:**
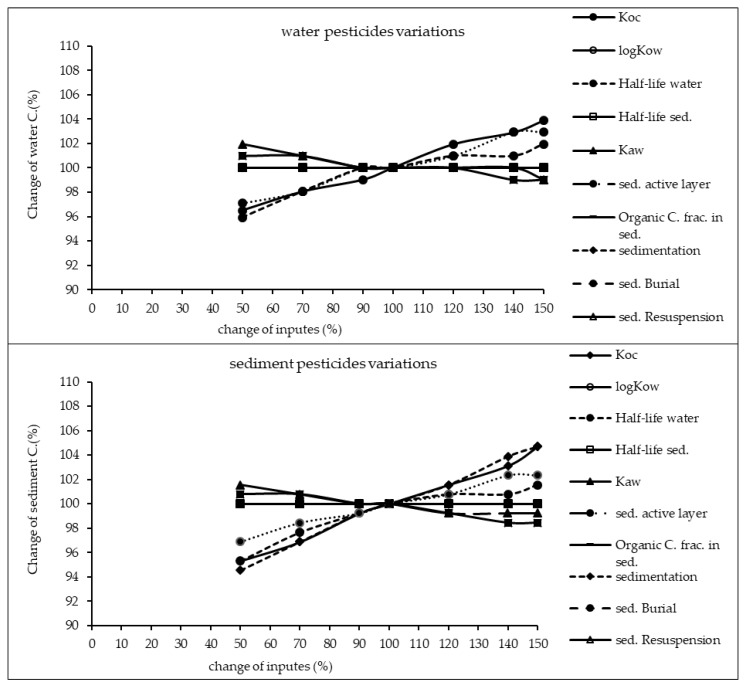
Results of the sensitivity analysis for α-HCH in Lake Naivasha.

**Figure 3:**
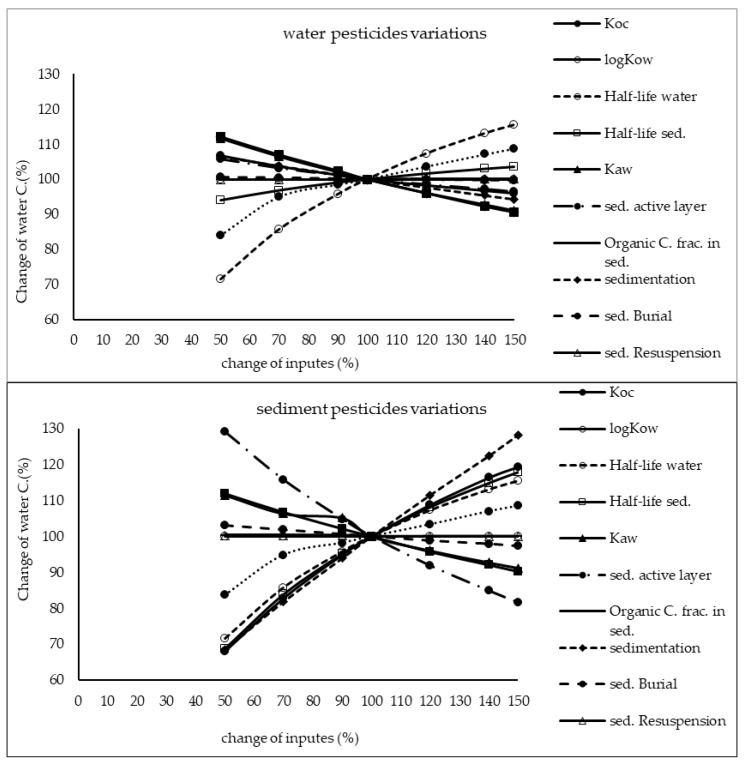
Results of the sensitivity analysis for methoxychlor in Lake Naivasha.

**Figure 4:**
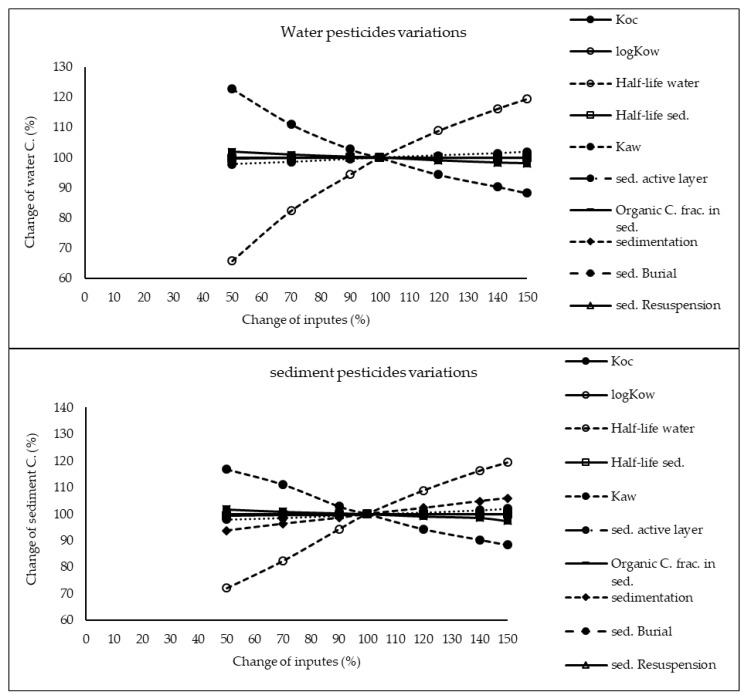
Results of the sensitivity analysis for endosulfan–sulfate in Lake Naivasha.

The results of sensitivity analysis of the parameters in the model are demonstrated in Figure 2, Figure 3 and Figure 4 and consequently the fitted values for the calibrated model are represented in Table 1 and Table 2. The results showed that the five most sensitive parameters were K_OC_, logKow, half-life of the pollutants in water, half-life of the pollutants in sediment, and K_AW_. It is noted that the uncertainties of some of these parameters, such as the half-lives were included, as they have been reported in different studies for other conditions. Therefore, defining a constant value was not reasonable; thus the calibration procedure allowed estimating a suitable amount in the reported ranges [39,40]. The sensitivity analysis showed that the percentage of the variations of the outputs (e.g., the concentration of the pollutants in either sediment or water) against the change of the input parameters was significantly different (Figure 2, Figure 3 and Figure 4). Moreover, the behaviors of the three studied pesticides residues were obviously different from each other. For example, the range of changes in the concentration of α-HCH residue was between 96% to 102%, while for methoxychlor and endosulfan–sulfate it was almost between 65% to 125%. This means that selecting the correct amount of parameters for modeling the methoxychlor has a higher effect on the results than the modeling of α-HCH. It is notable that mass balance models simplify the complex processes of the chemical’s fate. However, the results of such a model should be able to reflect the fate and the movement procedure of the pollutants [20]. Moreover, it is important to explain the scale of the uncertainties, which could be involved in the modeling results [20,43,44].

With regard to analyzing the sensitivity of different pesticide fate responses to the parameter changes, we assumed that the environmental dimensions were certain enough. Then, all of the environmental features were limited to some parameters such as sediment active layer, sedimentation and similar factors or chemical interaction factors in different media that can govern the fate and transformation of the chemicals [30]. The parameter of K_OC_ was found to be the key factor in the sensitivity analysis of α-HCH concentrations, both in the water and sediment, in which by increasing the amount of this parameter, the variations of the concentration were also increased. However, this parameter was of lower importance in changing the concentration of endosulfan–sulfate and methoxychlor residues in water and sediment. It was found that the half-lives of the chemicals also had an important role in governing the fate of all three studied pesticides, which could confirm that the interaction of the pollutants with the environment as well as their physicochemical properties had a significant effect on their existence in different environmental media. Moreover, from the variations of the pollutants’ concentrations against the physical parameters, it could be understood that some parameters such as sedimentation or sediment resuspension could influence the amount of pesticide residues in the sediment media than the aquatic phase. Therefore, it is concluded that for determining the fate of the pesticides in any one of the phases (e.g., water or sediment), estimating precise environmental parameter inputs was required.

**Figure 5:**
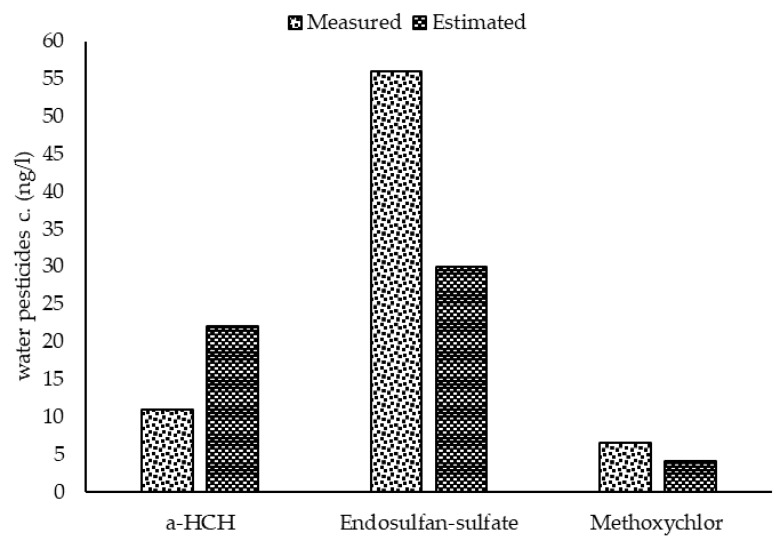
Comparison of the average measured and estimated concentrations of pesticide residues in the aquatic phase of Lake Naivasha.

With regard to the outcome of the sensitivity analysis, the model was calibrated based on the sensitive parameters. Comparing the average of measured concentrations to the results of the calibrated model (Figure 5) demonstrated that the model was calibrated reasonably. The statistical evaluation of the model calibration also showed an R-square of 0.65 and RMSE of 16.4, respectively. Therefore it can be concluded that the model predicts the concentration of the pollutants properly. Based on the study by Moriasi et al. [45], the model results with R-square between 0.30 to 0.65 is considered as satisfactory. In addition to this, using the passive sampling method helped calibrate the model with a high quality database and consequently produced more reliable results. 

**Figure 6:**
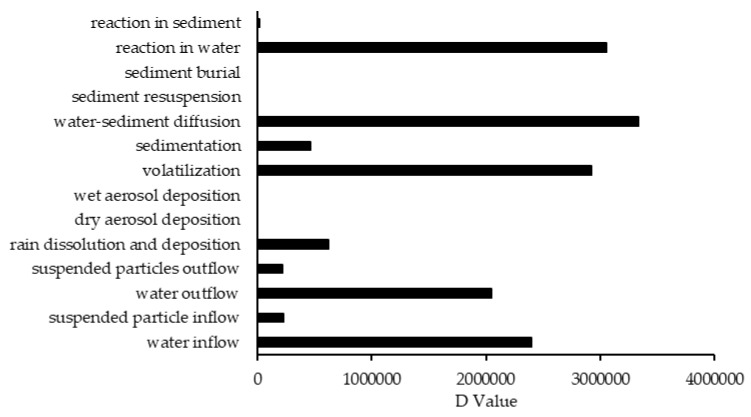
The D values of different processes that affect the fate of contaminants.

**Figure 7:**
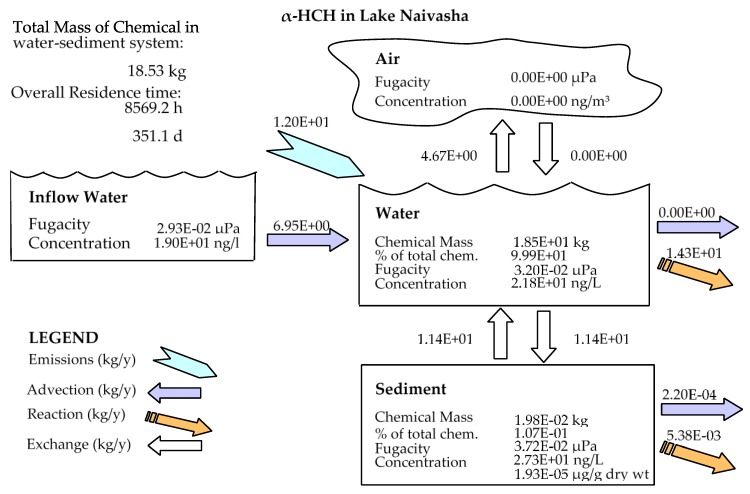

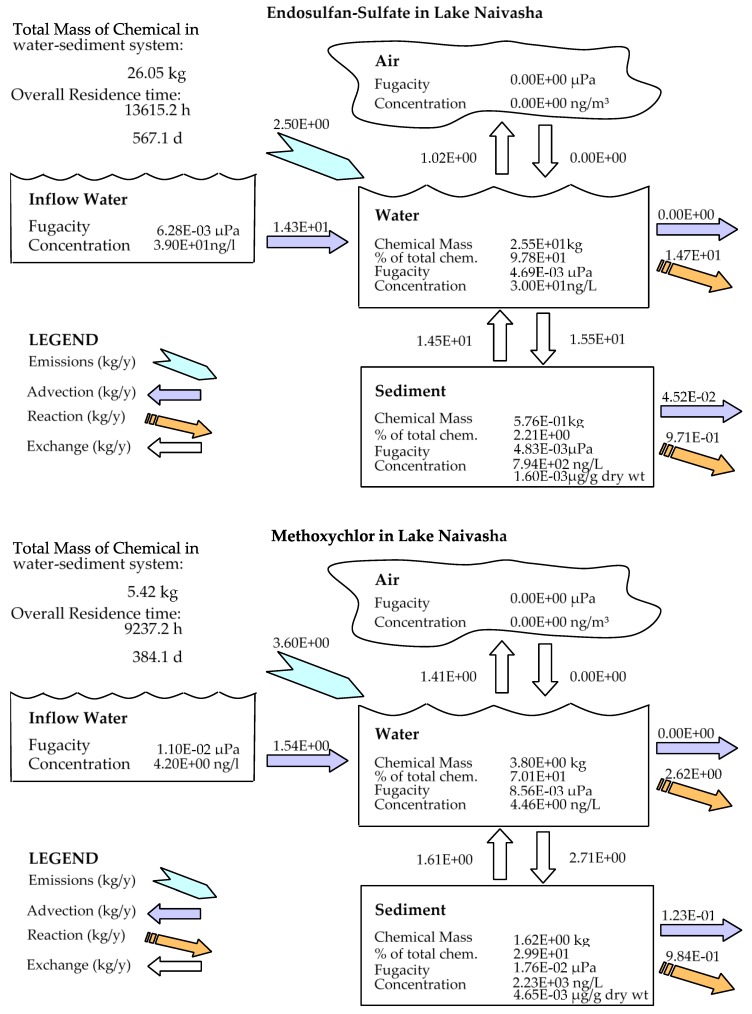
Mass balance diagram of different pesticides (α-HCH, endosulfan–sulfate and Methoxychlor) residues in the lake Naivasha.

In comparison to other studied lakes using QWASI (e.g., study by Mackay et al. [20]), lake Naivasha is a low volume and short retention time lake. Therefore, the modeling was based on well-mixed water assumption and there were no spatial differences in the lake chemical concentrations. While for the large lakes, the average concentration of the chemical has to be used and a multi compartment model that considers the variations has to be applied [20,46]. The D values (mol. Pa^−1^ h^−1^) are presented in Figure 6. These values are based on the fugacity amounts and show the process rate (mol/h) or the transformation of the chemicals [47]. In other words, the rate of the pollutants process in the environment is the product of these D values as well as the fugacity. However, with regard to the process that can affect the fate of pollutants, the results of mass balance modeling of the pesticides residues in the lake are presented in Figure 7. The results showed that there was a significant difference between environmental fate of methoxychlor and the other two kinds. These differences can be explained by the different half-lives and partitioning coefficients (e.g., K_OC_, LogK_OW_ and K_AW_) that influence the tendency of the pollutants for different levels of sedimentation, volatilization or suspension [48]. For example, the volatilization of methoxychlor is less due to the lower K_AW_, which is significantly less than α-HCH and endosulfan–sulfate (Table 1). The factor of K_OC_ is linked to the suspended compounds in the aquatic environment. It was found that methoxychlor had a higher amount of the sedimentation in which the K_OC_ value was more than 18 times larger than that of α-HCH and endosulfan–sulfate. Figure 7 shows the distribution of the chemicals among different media in which the amount of partitioning of α-HCH and endosulfan–sulfate to the sediment is similar (e.g., less than 1%) while it is significantly higher (e.g., almost 30%) for methoxychlor. This result can confirm the effect of the K_OC_ parameter on the fate and trend of the chemicals in the environment. The overall residence times of the chemicals were 351, 567 and 384 days for α-HCH, endosulfan–sulfate, and methoxychlor, respectively. The most important point of these residence times is that, although they are influenced by the half-life of the chemicals, they do not have a linear relation. This is because the residence time and consequently the deposition rates are governed, in addition to the half-life, by the parameters that influence the mass fractions and the loss processes [20]. 

Comparing the predicted amounts of pesticide residue concentrations in the water column and the sediment compartment showed that the OCP residues had different levels of pollutions (Table 3). It is noticeable that these amounts are the outcome of the combination of different factors such as physicochemical properties of the compounds, the emission rates and the environmental criteria. However, based on the available input data for the model, the results showed that methoxychlor had a mass fraction of almost 70% in the water column and almost 30% of the mass fraction in the sediment. In contrast, endosulfan–sulfate had the highest fraction in the water column (>97%) and just 2.2% in the sediment compartment. α-HCH also had the same situation of endosulfan–sulfate (e.g., 99% and less than 1% in water and sediment, respectively), and the absolute magnitude of this compound concentration falls between endosulfan–sulfate and methoxychlor. Generally, it can be concluded that in comparison to two other pesticides, methoxychlor had a higher affinity to sediments that can be mostly related to its high K_OC_ and low K_AW_ values, which highlights the role of these coefficients in the fate of pesticides residues. From the aspect of the emission rate also, there was a higher concentration of endosulfan–sulfate in the lake inflow, which comes from the lake catchment and resulted in more pollution in the lake. 

With regard to the pollution sources of the Lake Naivasha, it is notable that this lake is under the effect of the anthropogenic activities in the Naivasha catchment. In addition to this, the lake is also located beside Naivasha town. Then it can receive both hydrological and urban surface runoff and can be polluted by a number of point (e.g., sewage from the residential houses) or nonpoint (e.g., agricultural area) sources. Although importing and using OPCs in Kenya have stopped, there is still a potential for OCPs residues that can remain from the last or current usage [22]. There are some studies about OCPs pollution in the lake [21,49] that can confirm this statement. This study, which was based on the database of the passive sampling campaign [22], could highlight the existence of some OCPs residues in the water–sediment media of the lake. It is mentionable that there is not a long series of pesticides data in the water column or in the sediment to also do a time series evaluation. However, compared to conventional grab samplings, passive sampling allowed measuring chemicals in a low concentration, which was the strong point of the applied data of this modeling. 

Among the various physicochemical properties that contribute to the distribution and exchange of pollutants among the different media, air–water partitioning is important for more volatile compounds. For example, also pollutants that originate from air pollution can contribute to water pollution. This evaluation is based on their K_AW_ parameter and shows the importance of this factor to determine the affinity of the chemicals to the air or water bodies and vice versa [50]. Moreover, it is necessary to include the environmental properties also in the fate of the pollutant and the modeling results. For instance, the amount of evaporation loss has an inverse relation to the depth of water in which the less the water depth is, the more the evaporation rate increases [48]. Similarly, by increasing the content of suspended solids, more chemicals can be captured by the suspended particles and reduce the losses rate by evaporation. Moreover, by considering the environmental parameters (e.g., boundary conditions and the physicochemical properties of the pollutants), it can be understood that as these parameters vary, therefore, the results of the model also can be influenced. Organochlorine pesticides residues are mostly persistent in the environment and can remain in the nature (e.g., in aquatic phases) for a long period. However, their fate might be changed under the environmental variations that differ from the model predictions. In this study, QWASI allowed an insight into the fate process of the studied OCPs and helped with actual concentrations predictions. Therefore, it can be used to do risk assessment for the environmental exposure of pesticide residues. In different studies using QWASI model [20,30,51,52] or similar studies that applied the mass modeling to explore the fate of the chemicals, the capability of this modeling approach when combined with accurate field sampling and measurement was confirmed. In these studies, the sensitivity of the input data also was investigated and shows the importance of finding the most suitable parameters and selecting accurate amounts. In the current case study, the sensitivity analysis showed that K_OC_ had an important role in predicting the concentration, which was in harmony with the study by Whelan [30]. Finally, it is necessary to monitor data, by which the results of the modeling can be applied for long series.

## 4. Conclusions

The behavior of the pesticides residues and the controlling processes in the environment are governed by different physicochemical properties. Finding the relationship between the influencing criteria can help in understanding the fate of pollutants. However, this is a complex issue, and a model, in which all of the factors are included, is needed to explore the mechanisms that are related to partitioning, degradation features and environmental properties in the dynamic environment conditions. In this study, the QWASI model was used to find out the fate of some OCPs residues (namely α-HCH, endosulfan–sulfate and methoxychlor) in lake Naivasha. The lake was considered as a well-mixed environment in the modeling approach, and the data of the passive sampling campaign [22] as well as the environmental and physicochemical properties of the pesticides was used as the input data of the model. The physicochemical and the environmental data were also collected during the campaign or were found in the literatures. Because of the uncertainty that some of the parameters had, a sensitivity analysis and model calibration was accomplished. It was found that the model results were most sensitive to K_OC_ and the half-lives of the pesticides’ residues. In addition to these parameters, the coefficient values also had a meaningful effect on the output of the model and consequently the fate of the pollutants. All of the sensitive parameters were included in the calibration process in which the model could predict the fate of pesticide residues in the aquatic phase. Moreover, the results of modeling showed that because of the difference in the half-lives and the partitioning coefficients of the pesticides residues, there was a substantial difference between the fate of methoxychlor and α-HCH as well as endosulfan–sulfate. This difference was mostly expressed in volatilization from the water phase and sedimentation of the contaminants. The mass balance of the chemicals among different media showed the amount of partitioning of α-HCH and endosulfan–sulfate to the sediment was similar and was less than 1%, while this amount increased to almost 30% for methoxychlor.

Finally, the QWASI model allowed understanding the distribution of the chemicals among different environmental media. This output allows exploring the environmental exposure of tropical lakes to pesticide residues. Using reliable chemical data was also of high importance. In this study, applying data of the passive sampling method, which can help in measuring the chemicals at very low concentrations, was a strong point in modelling the fate of pesticides residues. 

## Figures and Tables

**Table 1:** Physicochemical properties of the pesticides used in the model calibration.

Compounds	α-HCH		Endosulfan–Sulfate		Methoxychlor	
Property	Initial Value	Fitted Value	Initial Value	Fitted Value	Initial Value	Fitted Value
K_OC_	3257	3151.71	1874	2771	35,000	49,292
logKow	3.9	3.72	3.6	3.8	4.5	5.08
Half-life water(hrs)	7884	8600	3600	5800	7200	8800
Half-life sed. (hrs)	9600	10,000	4270	6400	8500	10000
K_AW_	0.42	0.52	0.003	0.0054	0.000781	0.000781
Molar mass (g/mol)	290.83	290.83	422.9	422.9	345	345
Melting point (°C)	159	159	181.5	181.5	87	87
Vapor pressure (Pa)	0.0033	0.0033	0.000037	0.000037	0.0056	0.0056
solubility in water (mg/l)	2	2	0.22	0.22	1	1
Henry’s law constant	0.48	0.48	0.071	0.071	1.93	1.93

**Table 2:** Environmental properties used in the model calibration.

Property	Initial Value	Fitted Value
Surface area (m^2^)	145 × 10 ^6^	145 × 10 ^6^
volume (m^3^)	850 × 10 ^6^	850 × 10 ^6^
Mean lake depth (m)	6	6
Organic C fraction in sediment (g/g)	0.045	0.03
sed. active layer(m)	0.0075	0.005
Sediment deposition rate(g/m^2^.day)	1.815	1.21
Sediment burial rate(g/m^2^.day)	0.75	0.5
Sediment resuspension rate (g/m^2^.day)	0.06	0.04
Aerosol dry deposition rate(m/h)	10	30

**Table 3:** Predicted concentration of the OCPs compounds.

Pesticide	Con. In Water (ng/L)	Con. in sed. (ng/g dry wt.)	Mass in Water (kg)	Mass in sed. (kg)	Fraction in Water (%)	Fraction in sed. (%)
α-HCH	21.80	0.019	18.50	0.020	99	<1
Endosulfan–sulfate	30.00	1.600	25.50	0.560	97.8	2.21
Methoxychlor	4.46	4.650	3.80	1.620	70.1	29.9
